# Numerical Study of Crack Prediction and Growth in Automotive Wheel Rims

**DOI:** 10.3390/ma17051020

**Published:** 2024-02-22

**Authors:** Soufiane Montassir, Hassane Moustabchir, Ahmed El Khalfi, Sorin Vlase, Maria Luminita Scutaru

**Affiliations:** 1 Department of Mechanical Engineering, Faculty of Science and Technology, Sidi Mohamed Ben Abdellah University, Fez 30000, Morocco; ahmed.elkhalfi@usmba.ac.ma; 2 S3I Research Center, Ecole Arts et Métiers Campus of Rabat, Rabat-Salé 11100, Morocco; 3 Laboratory of Systems Engineering and Applications (LISA), National School of Applied Sciences, Sidi Mohamed Ben Abdellah University, Fez 30000, Morocco; hassane.moustabchir@usmba.ac.ma; 4 Department of Mechanical Engineering, Faculty of Mechanical Engineering, Transylvania University of Brasov, B-dul Eroilor 29, 500036 Brasov, Romania; svlase@unitbv.ro; 5 Romanian Academy of Technical Sciences, B-dul Dacia 26, 030167 Bucharest, Romania

**Keywords:** wheel rim, crack, failure, XFEM

## Abstract

Finite element analysis has become an essential tool for simulating and understanding crack growth. This technique holds significant importance in the field of mechanical engineering, where it finds wide application in the design and optimization of structural components and material properties. This work began with the identification of critical zones and estimated the number of load life repeats through fatigue analysis, specifically applied to automotive rims utilizing innovative finite element methods. To investigate crack behavior, we are used the Extended Finite Element Method (XFEM) with the volumetric approach to compute the Stress Intensity Factor (SIF). The results obtained by our study align closely with experimental tests in terms of detecting the critical zone where a crack can appear. Our findings contribute to the understanding of fatigue behavior in automotive rims, offering new insights into their structural integrity and performance under various load conditions.

## 1. Introduction

In a car, one of the most highly solicited parts is the rim. This part has a specific role in the vehicle, connecting the body and the tire parts and contributing to wheel rotation. This choice is not arbitrary but is based on economic factors and safety [1]. This element is the assembly by welding of a rim and a spoke. The wheel supports mechanical loads; it is therefore susceptible to damage and structural degradation such as cracking. These cracks can result from a variety of factors, including overloading and manufacturing defects. They appear on the surface of the wheel and can cause vibrations. In addition, they may propagate and weaken the structural integrity of the rim, increasing the risk of tire blowouts or even accidents.

**Figure 1:**
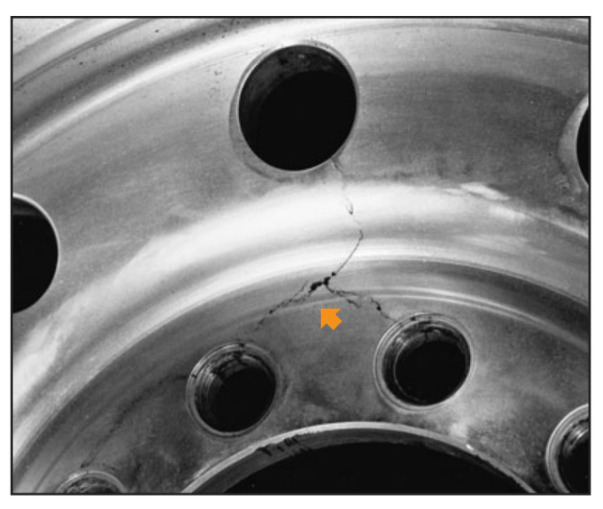
Crack growth near the rim hole illustrated by the position of the arrow.

In mechanical design, in order to properly design a structure, it is indispensable to take into account the phenomenon of cracking [2]. This subject has a major importance to researchers in this field, and a great deal of research is currently underway to find an effective way to predict crack behavior [3,4,5,6]. Figure 1 represents an example of crack growth near the hole of the wheel rim.

Many works study cracks, and some works may address the analysis of rims. In the railway field, Ref. [7] used a non-linear model to study crack propagation in the field of fatigue. Ref. [8] presented the spectral loading of a railroad wheel to treat the fatigue life and crack propagation deceleration. Ref. [9] studied the influence of temperature on a railway wheel to evaluate the fatigue failure mechanism. Ref. [10] investigated the importance of the residual stresses on the study of the fatigue life of a rail wheel. Regarding car wheels, Ref. [2] discussed the origins of these cracks but in the context of metallurgy. Ref. [11] used the finite element method to predict the fatigue failure of a heavy vehicle wheel.

To deal with the subject of cracking, there are specific techniques to predict crack initiation and growth. We can distinguish between two approaches: those based on the classical framework of Finite Element and those based on the Enriched Finite Element by introducing additional Degrees Of Freedom (DOF) and corresponding shape functions. The use of the Finite Element Method (FEM) includes some dedicated techniques, including remeshing or adaptive meshing, element elimination, method of cohesive elements or interface. Ref. [12] gives more details about these methods. The concept of the remeshing method [13] is centered on the geometric representation of the crack in the structure. The idea is to follow the propagation of the crack; i.e., for each stage of propagation, a new mesh explicitly describes the crack, and then a finer mesh must be created in the zones of interest close to the crack front and a coarse mesh for the rest. It is not widely used in industry, as it is generally too time-consuming to calculate [14]. The elimination of elements is another low-cost and extensively used method. The elements in which a mechanical quantity exceeds a specific criterion (maximum principal stress, von Mises stress, SIF, Integral J, etc.) are purely removed. The creation of this void represents the crack. This method does not allow for a true geometric representation of the crack. On the other hand, the mesh size has a significant impact on the numerical result.

**Figure 2:**
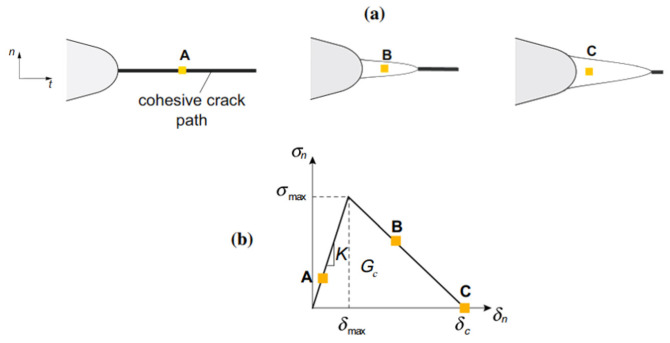
The cohesive method concept: (**a**) represents the cohesive zone concept, and (**b**) represents the bilinear cohesive stress-displacement relationship for quasi-brittle material [15].

The cohesive element method is based on the fact that the opening $\delta_{n}$ (the displacement jump) and a stress normal $\sigma_{n}$ to the crack are related by surface behavior as introduced in Figure 2 [15]. The energy dissipated $G_{c}$ to open the fracture is represented by the area under the usual stress-opening curve $\sigma_{n}=f(\delta_{n})$. It is still difficult to implement this method because of the necessary anticipated knowledge of the cracking path. To solve this kind of problem, a recent study on crack propagation using the X-FEM [16,17] was performed and highlights the power of the method. Ref. [18] provided a comprehensive survey of the XFEM literature. It is clearly observed that the use of traditional finite element methods to predict the behavior of crack propagation in a structure has a number of limitations.

Enrichment methods were then developed. Among the different enrichment methods, we find nodal enrichment where the enrichment degrees of freedom were added [19]. The methods based on the concept of unit partition [20] are called the nodal enrichment method, the Generalized Finite Element Method (GFEM) [21], and the XFEM [22,23,24,25].

The study of cracking problems aims to predict where or when the crack will propagate, i.e., to characterize fracture toughness. Consequently, we can find several fracture parameters: Crack Tip Opening Displacement (CTOD), Crack Tip Opening Angle (CTOA) and Stress Intensity Factor (SIF). The SIF is mostly used in the linear elastic fracture mechanics, and there are several types of software available to calculate this parameter. In this paper, ABAQUS CAE 2018 was used to model wheel rim failure with the XFEM and to calculate the SIF, but the limitation of this methodology is when choose the XFEM with allowing for crack growth and request the SIF through history output request, the fracture parameter (SIF) could not be obtained. Therefore, we propose to use the XFEM in order to obtain the normal stress distribution along the crack and to use the volumetric method [26] to calculate the SIF. This approach postulates that the fatigue phenomenon requires a certain physical volume; i.e., instead of using maximum stress to characterize the fracture, it is based on the effective stress. The determination of this stress was performed by determining the effective distance, which is strongly linked to the microstructure for low volumes of development of the fatigue process associated with high stress concentrations. Several works [27] show that this zone corresponds to the location where the relative stress gradient is minimum.

The organization of this research is detailed as follows: in Section 2, an overview of the theoretical background is described followed by the concept of the XFEM and the volumetric method. Then, there is a brief description of the numerical simulation proposed in this paper. In Section 3, an overview on the experimental data is presented. In Section 4, the results obtained are analyzed and discussed. Section 5 includes the conclusions that outline the main accomplishments of the paper.

## 2. Theoretical Background

In finite element analysis, Equation (1) is typically used to describe the displacement field.

(1) $$u\left(x\right)=\sum_{i=1}^{N}N_{I}(x)a_{i}$$

where $N_{I}$ and $a_{i}$ represent the shape functions and the degree of freedom associated. N is the number of the nodes.

Equation (1) is part of the conventional method where the treatment of the crack requires an adaptation of the mesh with the crack, and it generates singularities at the crack tip. To solve this problem, the XFEM extends this formulation by the use of the concept of unit partition. Equation (1) was further developed by adding other enrichment and degrees of freedom functions to capture the crack geometry and the crack tip. Equation (2) represents the displacement approximation for describing a crack in a body.

(2) $$u\left(x\right)=\sum_{i=1}^{N}N_{I}\left(x\right)\left[u_{i}+H\left(x\right)a_{i}+\sum_{j=1}^{4}F_{a}(x)b_{i}^{a}\right]$$

where $N_{I}$ is the finite element shape functions, $u_{i}$ is the regular degrees of freedom, *N* is the number of nodes, $a_{i}$ is the discontinuous degrees of freedom, $b_{i}^{i}$ is the *j*th singular degree of freedom, *H* represent the Heaviside enrichment function, and F represents the *j*th enrichment function.

### 2.1. Coupled Volumetric Method with XFEM

The aim of our paper is the study of fracture mechanics on wheel rims; this kind of study leads to the use of the SIF for the characterization of existing cracks. To quantify this parameter, in the case of crack propagation, it is required to develop a method because the ABAQUS CAE 2018 simulation software used in this paper does not enable the SIF to be calculated unless we consider a static crack. Our study is based on a new method, and the development of this method is based on the coupling of the XFEM and the volumetric method. The XFEM is well known in crack modeling, and it allows researchers how to overcome the big problem of stress singularity at the crack tip. Moreover, it is being adopted more and more by many commercial finite element software programs such as ALTAIR Radios, ABAQUS, ANSYS, etc. The volumetric approach is also showing its capability in this field [28]; it is assumed that the criterion for the initiation of failure depends on two parameters: the effective stress and the corresponding effective distance.

The methodology of our study consisted of plotting the stress distribution in the vicinity of the crack tip using the XFEM. Then, we used the volumetric approach to determine the effective stress (failure stress) and the effective distance. Finally, we deduced the SIF.

**Figure 3:**
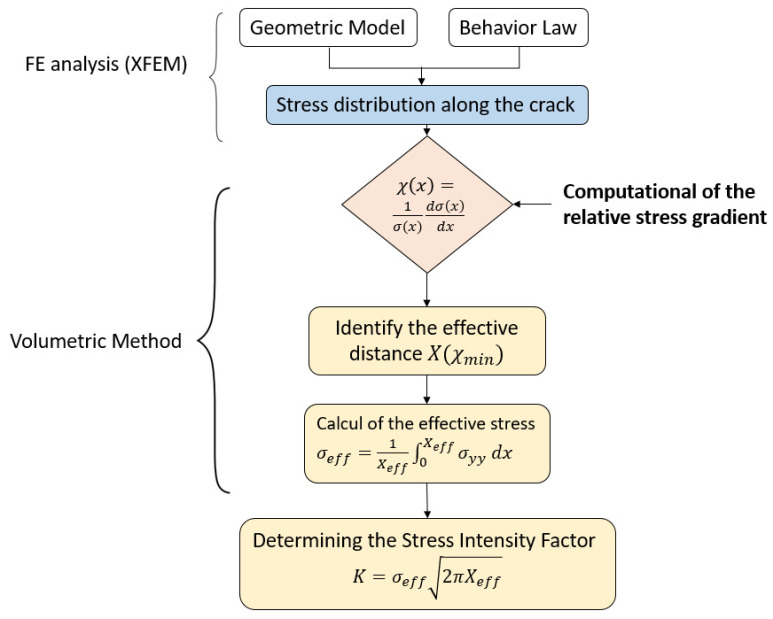
Steps to compute the stress intensity factor by coupling the X-FEM and volumetric method.

**Figure 4:**
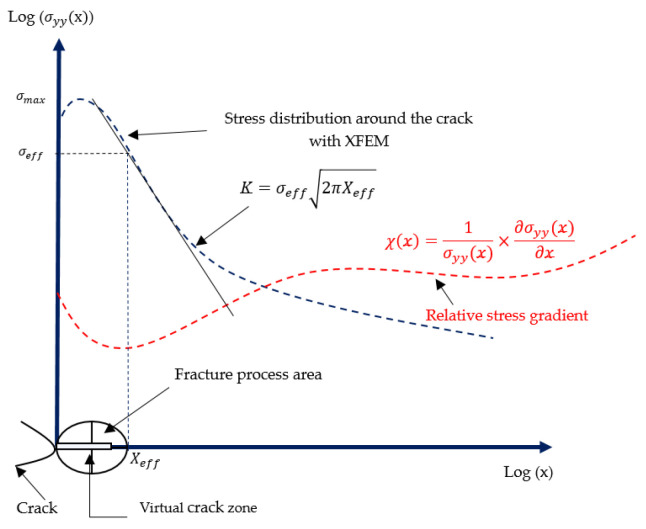
The process of the XFEM and the volumetric method.

To use the volumetric method, we followed the approach shown in Figure 3. We first introduced the geometric model with the behavior law, which aims to model the behavior of solids by an empirical law during deformation. We then plotted the stress distribution along the crack using the XFEM. Then, to calculate the parameters of the volumetric method (effective stress $\sigma_{eff}$ and effective distance $X(\chi_{min})$), we calculated the relative stress gradient. By combining the two curves (stress distribution and relative stress gradient), as represented in Figure 4, we obtained the effective distance, which represents the minimum of the curve, and by projecting this distance onto the stress distribution curve, we obtained the effective stress. At the end of this procedure, the SIF, represented by K, was obtained.

In Figure 4, we represented the combined curve obtained by the XFEM and the volumetric method. Where x, $\sigma_{yy}$, $\sigma_{max}$, $\sigma_{eff},X_{eff}\;and\;\chi (x)$ represent the distance along the crack, the normal stress along y, the maximal stress, the effective stress, the distance effective, and the relative stress gradient.

### 2.2. XFEM Setup

The XFEM has been proven as an effective tool for fracture analysis. In this paper, we propose to use the surface-based cohesive behavior with XFEM. In XFEM, the solution domain is extended to include the crack path, and the crack is modeled as a discontinuity in the material. In surface-based cohesive behavior, the material along the crack is modeled using a set of traction–separation laws. This approach has been widely used in various engineering applications, including the analysis of cracked structures [29,30] and failure prediction of composite materials [31]. The failure mechanism includes two parameters: the damage initiation and damage evolution criteria. The structural behavior was assumed to be linear elastic up to the initiation criterion being reached, and this parameter was based on stress or strain, both being available in ABAQUS CAE 2018 Software. Then, material degradation depends on the damage evolution, where two kinds of parameters define the evolution of damage: the first parameter consists of specifying the effective displacement at total failure, and the second involves specifying the energy dissipated because of the failure.

In this study, mode I was the mode of fracture considered, i.e., a crack opening where the tensile stress is normal to the crack plane. To study the fracture mechanism, we considered the maximum principal stress (MAXPS) and fracture energy $G_{c}$ as the two critical factors of failure that check crack initiation and toughness. The ultimate tensile strength was employed as limit value for the MAXPS value $\sigma_{\max\;ps}=\sigma_{ult}$, and the damage energy was estimated by $G_{c}=\frac{{K^{2}}_{IC}}{E}$ [32].

## 3. Overview of Experimental Data

### 3.1. Fatigue Test

**Figure 5:**
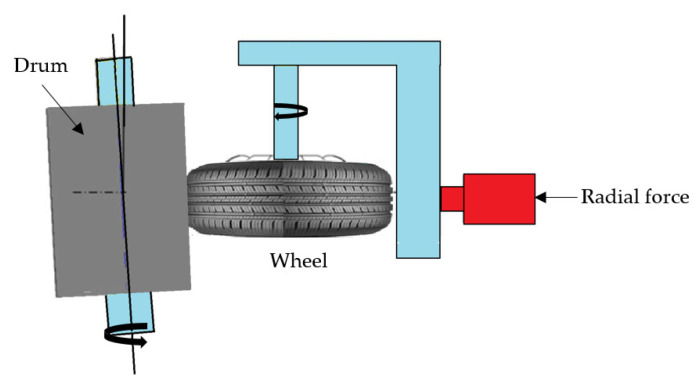
The fatigue test conditions [34].

To validate a prototype, the fatigue experiment test in Figure 5 is the most common solution in the manufacturing industry. The experimental results from the literature were utilized in this study [11,33].

### 3.2. Material Properties

Tensile tests and hardness measurements [11] were carried out to verify the mechanical characteristics of the wheel material shown in Table 1. The specimens were extracted from a wheel disc in accordance with DN 50125, and they were used in the tensile tests in accordance with DINEN10002-1.

## 4. Numerical Analysis, Results and Discussion

### 4.1. XFEM Model

**Figure 6:**
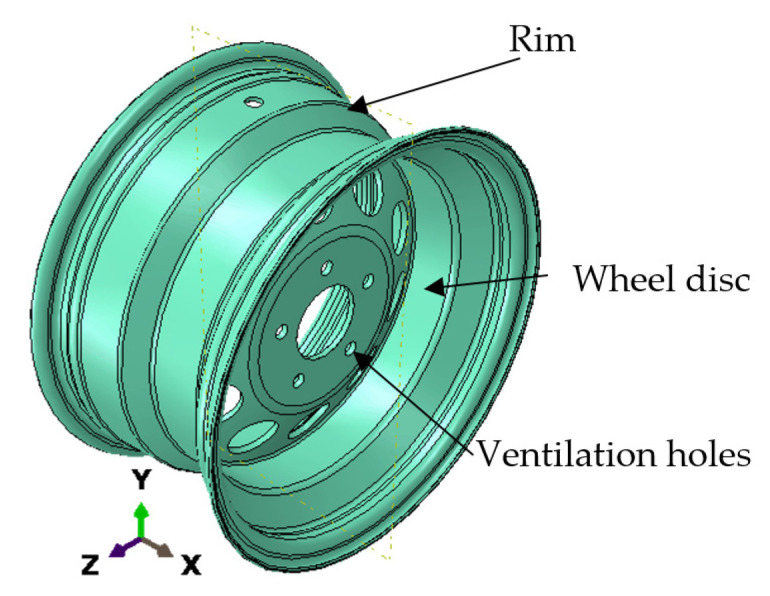
The model of the wheel rim.

The wheel disc and the rim flag are the basic parts of a tubeless wheel Figure 6. The assembly process used to join these two components is welding. Due to stress concentration in the ventilation holes of the wheel disc [34], a crack may appear in this area. The sample with no predefined crack was modeled in Abaqus and Fe-safe. They are commercial software; the first is a software suite for finite element analysis and computer-aided engineering, and the second is software used for fatigue analysis. The wheel was modeled as elastic material. We considered in this paper a model geometry that represents a generic and realistic approach to commercial models with the dimensions represented in Table 2.

The model was imported on ABAQUS. The reference points were defined on the wheel for the applications of loads. The surface-based coupling constraint in ABAQUS was used to provide coupling between the reference point and the corresponding surface.

### 4.2. Boundary Conditions

**Figure 7:**
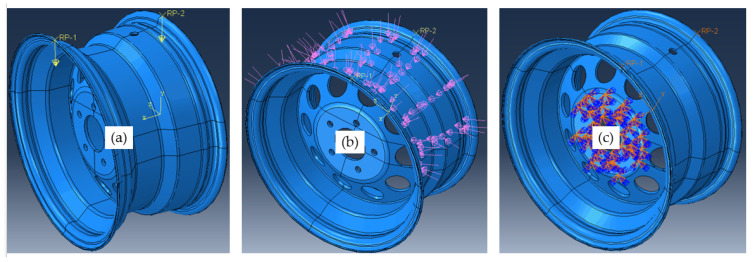
The boundary condition representation: (**a**) is the effect of the inflation area of the tire; (**b**) is the contact between tire bead seat rim and (**c**) is the fixed boundary conditions.

The finite element analysis was carried out by taking into account the radial test load $F_{r}$ and the impact of the tire inflation pressure $p_{i}$. In order to make the simulation closer to reality, we took the conditions that were in the experimental test. Figure 7 represents the configuration adopted in this simulation. For the loads, they were distributed as follows: to apply the effect of the inflation area of the tire, the force $F_{r}$ is represented as shown in Figure 7a, and to represent the contact between the tire bead and seat rim, we applied a P force as represented in Figure 7b. The wheel rim was clamped on out bolt holes as shown in Figure 7c.

According to the test study of [11], we adopted a tire pressure of $P_{i}=$ 1 MPa and a nominal test load of P= 78 KN.

### 4.3. Mesh Detail

**Figure 8:**
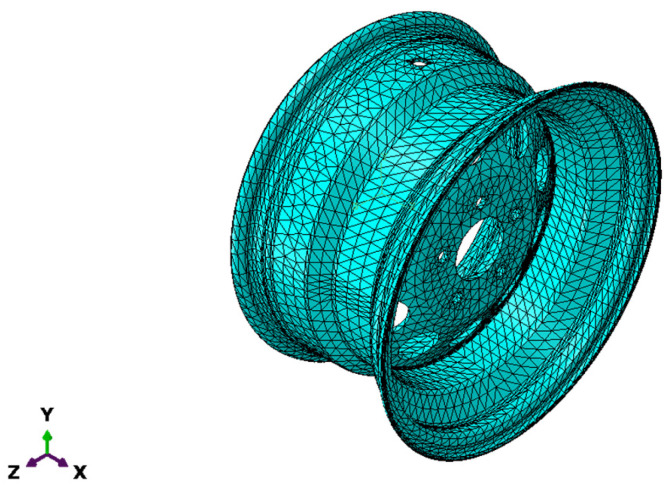
The model representing the wheel rim.

**Figure 9:**
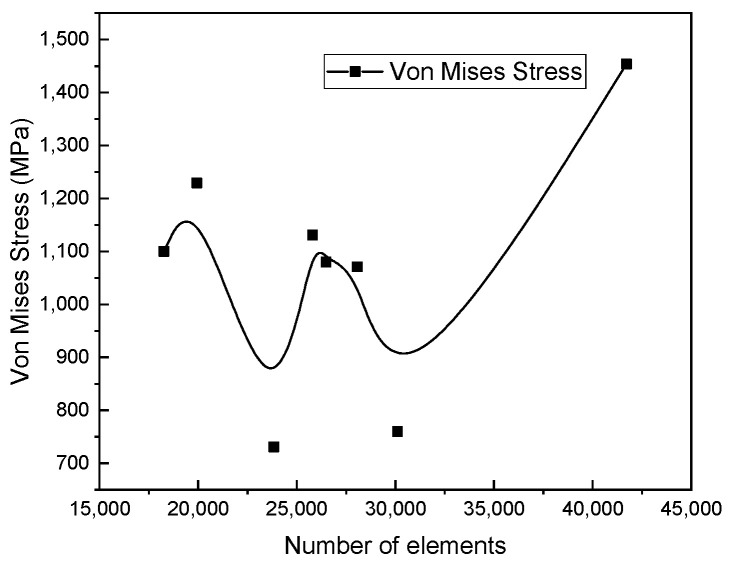
Mesh sensitivity with different elements.

In the mesh part, the rim was partitioned to have a finer mesh at the ventilation holes. Therefore, the partitioned parts have fine mesh and the rest of the structure a coarse mesh. Figure 8 visualizes the distribution of the mesh within the model. C3D10 is the element type that was used to represent the wheel rim as a three-dimensional solid continuum; it is suitable for simulating complex geometric shapes with curved surfaces. To enhance the accuracy of the outcome, the solid’s mesh was refined. Mesh sensitivity was performed to select an adequate mesh, which can produce acceptable simulation results. Figure 9 shows that as the number of elements increases, the von Mises stress changes. This is because changing mesh size affects the XFEM model [35] and a different solution was obtained. An optimum mesh size of 15 mm was adopted in this work in order to study the cracking that occurs in the ventilation holes. The result was stabilized with 49,862 as the total number of nodes and 25,810 as the number of elements.

### 4.4. Model Calibration

Material behavior with fracture stress and fracture energy as damage parameters was modeled using elasticity. To model the evolution of damaged material to an open crack, a linear traction–separation law was employed.

This work shows for the first time the use of the XFEM with the volumetric method to study the behavior of the crack in the wheel rim. Calibrating the Maximum Principal Stress (MAXPS) and fracture energy ($G_{c}$) is important for this proposed technique. There are some works that calibrate those parameters in the pipe geometry case. Ref. [17] employed a comparative method to calibrate the Maximum Principal Strain (MAXPE) and $G_{c}$ parameters, and this calibration was subsequently used to accurately predict the pipe’s tensile strain capacity based on experimental data. Similarly, [36] calibrated the MAXPE and $G_{c}$ damage parameters using a load versus Crack Mouth Opening Displacement (CMOD) curve. Ref. [17], on the other hand, utilized Crack Tip Opening Displacement (CTOD) curves from Single-Edge Notched Bending (SENT) tests to calibrate the Maxps and $G_{c}$ damage parameters.

Due to the lack of experimental results that deal with cracking in the wheel rim geometry, we proposed using the MAXPS parameter and fracture energy $G_{c}$ as the fracture parameter. For the MAXPS parameter, the ultimate tensile strength $\sigma_{ult}$ of the S355 steel was employed, and for the damage energy, it was based on the fracture toughness $K_{IC}$. Ref. [37] studied the fracture toughness of the material used in this study using Equations (3) and (4).

(3) $$K_{Ic}=\frac{P_{f}}{D^{3/2}}\times \left[1.72\left(\frac{D}{d_{eff}}\right)-1.27\right]$$

(4) $$d_{eff}=D-2(a_{m}+a_{f})$$

where $d_{eff}$ is the effective diameter, *D* is the diameter of unnotched section, $a_{m}$ is the machined notch depth, $a_{f}$ is the fatigue pre-crack and $P_{f}$ is the fracture load.

According to Equation (3), the fracture toughness $K_{Ic}$ varies from 35.78 Mpa$\sqrt{m}$ to 40.4 MPa$\sqrt{m}$ [37]. Therefore, the fracture toughness obtained through experimental means, on average, is 38.1 MPa$\sqrt{m}$.

### 4.5. Fatigue Analysis

**Figure 10:**
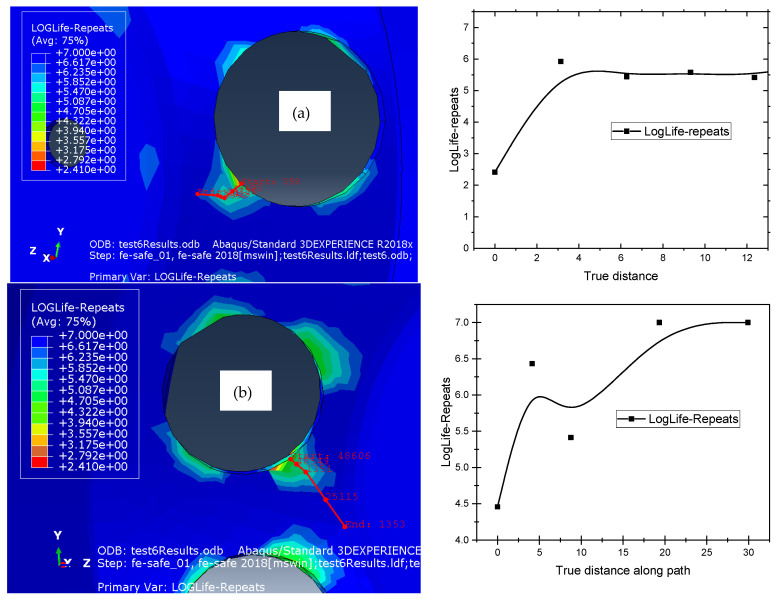
Fatigue study for outer (**a**) and inner (**b**) surface.

To estimate rim fatigue life within the $10^{5}\;and\;10^{6}$ cycles, Fe-safe and Abaqus were used. We generated a result file, output database (.odb), from Abaqus, and then we imported it on Fe-safe, and this format is compatible for the software packages, because we based it on the distribution of the von Mises stress, and in Fe-safe software 2018, we proposed to use the SN curve to obtain reliable results [38]. This curve was constructed with ${N=10}^{2}$ and corresponded to S=564.728 MPa, and $N=10^{6}$ corresponded to S=133.93 MPa. The fatigue life of the processed wheel disc material was determined using the biaxial stress–life approach [39]. The fatigue analysis was performed according to three models; Gerber, Goodman and R ratio SN curve Table 3. Figure 10 illustrate the result of the fatigue analysis with the diagram of LogLife-repeats versus true distance (distance shown in red on the left of the figure) for the inner and outer surface of the rim wheel. 

### 4.6. Comparison between the Proposed Method and Experimental Method

**Figure 11:**
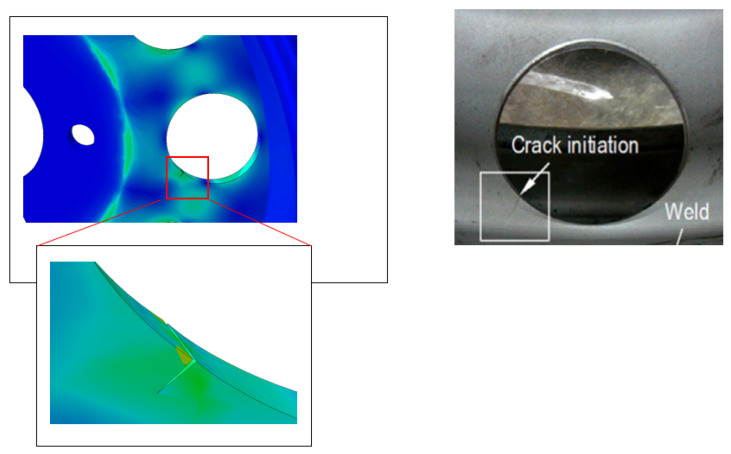
The comparison between the XFEM model and experimental test.

**Figure 12:**
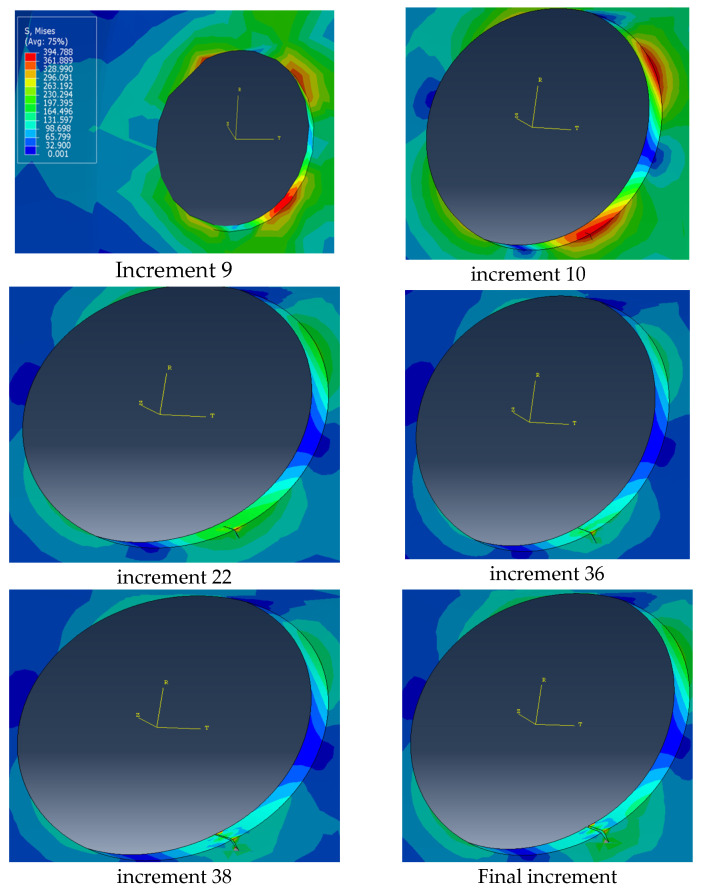
Different increments of degradation surface.

The analysis performed in this paper is a static analysis using ABAQUS 2017 software. The numerical simulation showed that the stress is concentrated in specific areas on the air ventilation holes and, in these regions, a crack began to initiate. The numerical results obtained correspond well with the experimental test in Figure 11. In this study, the initiation was performed in regions with a high stress concentration, after increment 10, the first stage of propagation occurs. The different stages of surface degradation are represented in Figure 12. It is clear that in the area of concentrated stress (in red), a crack has begun to appear, and with increments, we can see the crack opening.

### 4.7. Stress Intensity Factor

**Figure 13:**
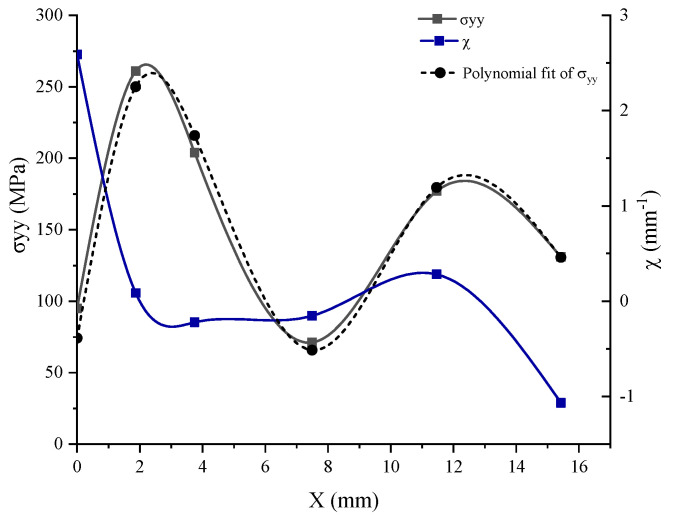
Diagram of the elastoplastic stress, the polynomial fit and the gradient of $\sigma_{yy}$.

To compute the stress intensity factor, the volumetric method was chosen in Figure 13. Based on the finite element model, the elastoplastic stress around the crack was obtained and the curve has a maximal value of $\sigma_{max}=261.017$ MPa at $X_{max}=1.867$ mm, which is close to the result obtained with ANSYS by using the classical finite element method to study the stress analysis [11]. Then, to apply the concept of the volumetric method, we needed to plot the polynomial fit of the $\sigma_{yy}$. To determine the parameters of this method and the effective stress and distance, we used the distribution of the relative stress gradient χ. The equations mentioned in Figure 3 enabled us to calculate the effective stress $\sigma_{eff}=$ 250 MPa and the effective distance $X_{eff}=3\;mm$.

In our study, the stress intensity factor SIF or K can be estimated to provide a failure criterion for the crack growth by using the last formula mentioned in Figure 3 and the results based on the volumetric parameters. It is represented as follows:

(5) $$K=250\sqrt{2\pi \times 3\times 10^{-3}}=34.31\;MPa\sqrt{m}.$$

## 5. Discussions

By combining the finding of the XFEM and Fe-safe, the fatigue life of the wheel disc was estimated. Based on the results obtained from fatigue analyses, the critical region of the ventilation hole was predicted to be where crack initiation may occur within N = 2.69 × 10^5^ load life repeats by using the R ratio SN curve model, and for the Gerber and Goodman models, the load life repeats are 1.45 × 10^5^ and 5.25 × 10^4^, respectively. Furthermore, the result obtained by the FEM analysis and the radial test are N = 6.45 × 10^5^ and N = 1.04 × 10^6^, respectively. The result obtained by the Goodman model is a little further away than the normal stress R ratio SN curve and Gerber models, so it can be said that the wheel rim can be damaged in this interval N = 1.45 × 10^5^ – 2.69 × 10^5^. In comparison with the literature results, our values are a little similar, and this difference is due to the fact that the geometric model used in our study is not the same as the literature. Therefore, we conducted a comparative study with the literature and experimental tests to assess the reliability of this method, and we found that the areas with stress concentration are the same, meaning that in most cases of wheel disc deterioration, the crack initiates from the ventilation hole zone.

In order to be able to study the fatigue life of the wheel disc, stress-based methods were adopted in this study. The Loglife repeats were estimated at the critical region in the outer and inner surfaces, and the results of the fatigue studies for the two surfaces are given in Figure 10. Figure 10 shows the Fe-Safe simulation result, and in the critical areas we identified, we plotted the Loglife repeats near these regions. From these results, we were able to estimate the number of cycles where there is a stress concentration, i.e., the critical zone, and we presented the results along the distance, which is near this zone. These results show that the inner surface in Figure 10a has a longer life than the upper surface in Figure 10b. If we only take the point on the edge of the hole for the inner surface, we have 4.5 of Loglife repeats that correspond to $10^{4.5}$ load life repeats, and for the outer surface, we have 2.4 of LogLife repeats that correspond to $10^{2.4}$. According to the model results, it can be mentioned that the initiation of the crack will start from the outer surface, and after cycles, it can appear on the inner surface.

The fatigue study allowed us to estimate the load life repeats before failure and predict the zone that will be most resistant to stresses. The novelty of this paper is that we did not create or assume the existence of a crack, but rather based on the study of stresses and fatigue, we only indicated the zone where a crack could appear and then focused on this to visualize the crack opening. The results show that in the zone where there is a concentration of stress, a crack initiation began as shown in the Figure 12. The results obtained in this work provide corroboration for our earlier findings derived from the fatigue study, establishing a crucial link between the location identified as the critical zone and the emergence of a crack. This significant correlation underlines the validity of our previous research and reinforces the importance of the identified critical zone in understanding the cracking process. The crack appeared on the outer surface and subsequently propagated to the inner surface.

Crack growth is a critical aspect of fracture mechanics, as it helps in understanding the behavior of materials under stress. To achieve this, the use of fracture parameters is essential, as they enable the behavior of cracks to be quantified. However, in cases where the propagation path of a crack is to be estimated, the calculation software may not support the calculation of the fracture parameter. To address this problem, we proposed the use of the volumetric method. This approach involves coupling the distribution of constraints obtained by the XFEM with the stress gradient. The result is the determination of the SIF, K= 34.31 MPa$\sqrt{m}$, which is crucial in predicting the propagation of a crack. According to the experimental result of the fracture toughness of the material used in this paper $K_{ic}=$ 38.1 MPa$\sqrt{m}$, we can say that the SIF value does not exceed the fracture toughness, which implies that the crack will not propagate rapidly, but, on the other hand, the wheel may have low crack propagation under cyclic loading.

Our proposed method is particularly useful in cases where the calculation of the fracture parameter is not feasible. Additionally, it provides a means of estimating the propagation path of a crack, which is essential in designing and assessing the safety of structures. In conclusion, the use of the volumetric method offers a promising approach to the estimation of crack propagation. Further research in this area could lead to the development of more robust and accurate methods for predicting crack behavior.

## 6. Conclusions

In this research paper, we have presented an innovative finite element-based method for studying the cracking of automotive rims due to radial testing. Our approach involves the use of Fe-Safe to estimate the number of cycles in a fatigue study and determine the critical zones where stress concentration occurs. We then used the XFEM to successfully predict the appearance of cracks on the outer surface, as predicted by the fatigue test.

We calculated the fracture parameter using the XFEM and the volumetric method, which enabled us to accurately estimate the stress intensity factor (SIF). A significant innovation of our study is the introduction of a new technique, which does not rely on the presence of a pre-existing physical crack, but instead focuses on the prediction of crack initiation. In parallel, we carried out fatigue calculations to validate our results, drawing on previous research, in particular that of [11], as a comparative reference.

Our study extended to the precise location of stress concentration zones, as shown in Figure 10. When analyzing lifespan, the results obtained from R ratio SN curve and Gerber models are close to the literature results. Several factors may affect the results. Firstly, rim dimensions, as variations in size can have an impact on stress distribution. In addition, the numerical methods employed could explain some of the variations observed. Finally, the choice of fatigue parameters, which are intrinsically complex, may introduce additional variability into the results.

In conclusion, this research paper provides an important contribution to the field of fracture mechanics, offering a novel approach to predicting rim cracking and providing insights into critical areas. Our proposed method has the potential to inform the design and testing of safer, more resilient automotive wheel rims, and we hope that future research in this area will further refine and improve our approach.

## Figures and Tables

**Table 1:** The mechanical characteristic of the S355MC steel [11].

Young’s modulus [GPa]	210
Poisson’s ratio	0.3
Yield stress [MPa]	374.51
Ultime stress [MPa]	522.57
Maximum Elongation [%]	24.75

**Table 2:** The wheel rim dimensions.

Rim Diameter	381 mm
Rim Width	203.2 mm
Backspace	76.2
Number of bolts/stud holes	6 × 139.7 and 5 × 114.3

**Table 3:** The fatigue analysis based on the Gerber model, Goodman model and the normal stress R ratio SN curve theory with the literature and test results.

Gerber	Goodman	R Ratio SN Curve	FEM Analysis [11]	Radial Test [11]
1.45 × 10^5^	5.25 × 10^4^	2.69 × 10^5^	6.45 × 10^5^	1.04 × 10^6^

## Data Availability

Data are contained within the article.
